# Improvement of Neurovascular Imaging Using Ultra-High-Resolution Computed Tomography Angiography

**DOI:** 10.1007/s00062-023-01348-1

**Published:** 2023-10-13

**Authors:** Felix A. Ucar, Marius Frenzel, Andrea Kronfeld, Sebastian Altmann, Antoine P. Sanner, Mario Alberto Abello Mercado, Timo Uphaus, Marc A. Brockmann, Ahmed E. Othman

**Affiliations:** 1 Department of Neuroradiology, University Medical Center Mainz, Langenbeckstr. 1, 55131 Mainz, Germany; 2 Department of Computer Science, Fraunhofer IGD, Technical University Darmstadt, Fraunhoferstraße 5, 64283 Darmstadt, Germany; 3 Department of Neurology, University Medical Center Mainz, Langenbeckstr. 1, 55131 Mainz, Germany

**Keywords:** Computed tomography, Computed tomography angiography, Resolution, Cerebral arteries, Image enhancement

## Abstract

**Objective:**

To evaluate diagnostic image quality of ultra-high-resolution computed tomography angiography (UHR-CTA) in neurovascular imaging as compared to normal resolution CT-angiography (NR-CTA).

**Material and Methods:**

In this retrospective single-center study brain and neck CT-angiography was performed using an ultra-high-resolution computed tomography scanner (*n* = 82) or a normal resolution CT scanner (NR-CTA; *n* = 73). Ultra-high-resolution images were reconstructed with a 1024 × 1024 matrix and a slice thickness of 0.25 mm, whereas NR-CT images were reconstructed with a 512 × 512 matrix and a slice thickness of 0.5 mm. Three blinded neuroradiologists assessed overall image quality, artifacts, image noise, overall contrast and diagnostic confidence using a 4-point Likert scale. Furthermore, the visualization and delineation of supra-aortic arteries with an emphasis on the visualization of small intracerebral vessels was assessed using a cerebral vascular score, also utilizing a 4-point Likert scale. Quantitative analyses included signal-to-noise ratio (SNR), contrast-to-noise ratio (CNR), noise and the steepness of gray value transition. Radiation exposure was determined by comparison of computed tomography dose index (CTDIvol), dose length product (DLP) and mean effective dose. Interrater agreement was evaluated via determining Fleiss-Kappa.

**Results:**

Ultra-high-resolution CT-angiography (UHR-CTA) yielded excellent image quality with superior quantitative (SNR: *p* < 0.001, CNR: *p* < 0.001, steepness of gray value transition: *p* < 0.001) and qualitative results (overall image quality: 4 (Inter quartile range (IQR) = 4–4); *p* < 0.001, diagnostic confidence: 4 (IQR = 4–4); *p* < 0.001) compared to NR-CT (overall image quality: 3 (IQR = 3–3), diagnostic confidence: 3 (IQR = 3–4)). Furthermore, UHR-CT enabled significantly superior delineation and visualization of all vascular segments, from proximal extracranial vessels to the smallest peripheral cerebral branches (e.g., UHR-CTA PICA: 4 (3–4) vs. NR-CTA PICA: 3 (2–3); UHR-CTA P4: 4 (IQR = 3–4) vs. NR-CTA P4: 2 (IQR = 2–3); UHR-CTA M4: 4 (IQR = 4–4) vs. NR-CTA M4: 3 (IQR = 2–3); UHR-CTA A4: 4 (IQR = 3–4) vs. NR-CTA A4: 2 (IQR = 2–3); all *p* < 0.001). Noteworthy, a reduced mean effective dose was observed when applying UHR-CT (NR-CTA: 1.8 ± 0.3 mSv; UHR-CTA: 1.5 ± 0.5 mSv; *p* < 0.001).

**Conclusion:**

Ultra-high-resolution CT-angiography improves image quality in neurovascular imaging allowing the depiction and evaluation of small peripheral cerebral arteries. It may thus improve the detection of pathologies in small cerebrovascular lesions and the resulting diagnosis.

## Introduction

Noninvasive techniques have become highly relevant in neurovascular imaging, and computed tomography angiography (CTA) nowadays represents a key component therein. Computed tomography (CT) is of crucial importance in neuroradiological imaging and has excelled in clinical routine due to its ubiquitous availability in cases of emergency while maintaining high diagnostic accuracy. Main goals in neurovascular imaging are rapid volume coverage with the ability to detect pathologies in small diameter vessels at high sensitivity and specificity. Normal resolution CTA (NR-CTA) has been the working horse in neurovascular imaging for decades, but it is generally limited in the delineation of anatomical small diameter vessels and small cerebrovascular lesions due to its limited spatial resolution. The most recent developments in CT technology and reconstruction algorithms enabled radiologists to acquire image data with increased, ultra-high-resolution [1]. There are currently two methods for generating ultra-high-resolution CTAs: photon counting technology [2, 3] and the energy-integrated CT system with very small detector elements investigated here. Recently, one of the first UHR-CTs with advanced detector elements (element size, 0.25 mm at isocenter, superfine detector grids) and a small focal spot size (smallest size 0.4 × 0.5 mm) was introduced and implemented into the clinical routine to further improve neurovascular imaging. First technical reports have demonstrated the capability of UHR-CT to improve image quality in temporal bone imaging [4], coronary artery imaging [5, 6] and thoracic imaging [7, 8]; however, the capability of UHR-CT in comparison to NR-CT in neurovascular imaging has not been investigated thoroughly. With this novel technology at hand we hypothesized that the UHR-CTA could provide superior image qualities compared to NR-CTA, hereby adding value to the visualization of smaller vessels and cerebrovascular lesions. Hence, the aim of this study was to comprehensively evaluate the image quality metrics and the diagnostic possibilities of vascular imaging of UHR-CTA compared to NR-CTA.

## Material and Methods

### Study Population

This retrospective study was approved, and written informed consent was waived by our local ethics committee. All methods were performed in accordance with the 2008 version of the Declaration of Helsinki.

**Fig. 1:**
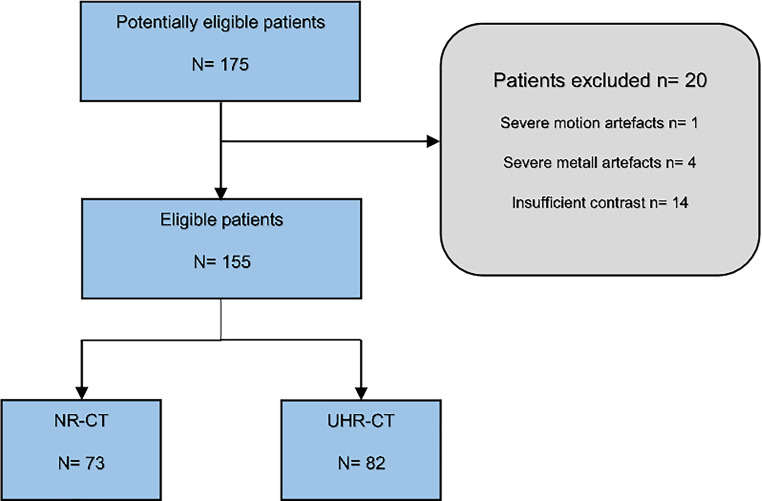
STARD flowchart of eligible patients and inclusion/exclusion process

The study population included consecutive adult patients who presented with acute neurologic symptoms and received a diagnostic UHR-CTA or NR-CTA at our department from September 2021 to December 2022. Exclusion criteria were distinct motion artifacts, beam-hardening artifacts and an insufficient contrast due to venous overlay (Fig. 1).

### CT Examination and Scanner Systems

The UHR-CT system (Aquilion Precision; Canon Medical Systems Corporation; Japan) featured a detector element size of 0.25 mm, a focal spot size of 0.4 × 0.5 mm, a beam collimation of 0.25 × 160 mm rows with 1792 channels and a spatial resolution of up to 50 lp/cm. The clinically optimized parameters of the CTA protocol consisted of a smallest focal spot size of 0.8 × 1.3 mm, a detailed pitch of 0.569, and a tube voltage of 120 kV, a field of view of 180 mm, and a rotation time of 0.35 s per rotation. The acquired images were reconstructed using a 1024 × 1024 matrix.

The NR-CT scanner featured a detector element size of 0.5 mm, a beam collimation of 0.5 × 32 mm rows, and a maximum spatial resolution of 18 lp/cm. The parameters of the CTA protocol consisted of a focal spot size of 0.8 × 1.3 mm, a helical pitch of 0.844, a tube voltage of 120 kVp, a field of view of 180 mm, and a rotation time of 0.5 s per rotation. The focal spot size was 1.6 × 1.4 mm. Images were reconstructed using a matrix of 512 × 512.

Both CT scanners used the auto-exposure control (AEC) with automated current adjustment to generate a radiation dose as low as reasonably achievable.

For both protocols the nonionic contrast agent iopromide (Ultravist-370; Bayer Healthcare, Germany) was injected using a high-pressure syringe system for advanced clinical CT-imaging procedures (Accutron CT‑D; Medtron; Saarbrücken, Germany) and 65 ml of the prewarmed contrast agent was administered via a peripheral venous catheter in the antecubital fossa at a flow rate of 5 ml/s, immediately followed by a 60 ml saline bolus.

According to our standards and for sufficient comparability, a monitoring region of interest (ROI) was placed in the ascending aorta to consolidate arterial contrast while minimizing venous contamination. Timing was optimized using automatic trigger acquisition and enhancement threshold selection. Acquisition started after reaching a threshold of 180 HU (Hounsfield units) in the aortic arch.

### Image Reconstruction and Processing

The axial source images of UHR-CTA (slice thickness 0.25 mm) were reconstructed in 3 planes with slice thicknesses of 0.25 mm, 1 mm and 3 mm, respectively and a reconstruction matrix of 1024 × 1024 pixels. The adaptive iterative three-dimensional dose reduction tool AIDR 3D standard (Canon Medical Systems Corporation) with an FC41 soft tissue kernel adapted to UHR was used as a reconstruction algorithm.

The NR-CTA source images with slice thicknesses of 0.5 mm were reconstructed in 1 mm in coronal and sagittal reformation from a 512 × 512 pixels matrix using a filtered back projection with an FC04 soft tissue kernel.

### Qualitative Evaluation

The acquired data were transferred to a workstation equipped with additional modules for neuroradiological vascular analysis (Sectra Workstation, Linköping, Sweden).

Overall image quality, diagnostic confidence, overall contrast, noise and artefacts were evaluated by three radiologists (blinded data, A.M, and A.S: both 5 years of experience in neurovascular imaging and A.O: more than 10 years of experience) on a 4-point Likert scale with 4 being best (Table 1). For a dedicated evaluation of cerebral vasculature, we developed a cerebral vessel score (Table 2) which was applied to the following vessels: internal carotid artery (ICA), vertebral arteries (VA), posterior inferior cerebellar artery (PICA), anterior inferior cerebellar artery (AICA), superior cerebellar artery (SCA) and the anterior, middle and posterior cerebral artery (ACA, MCA, PCA, respectively) and their subsegments. For training and reaching a standardized consensus regarding the 4‑point Likert scale, images from 20 patients, who were not included in the study were used.

**Table 1:** Likert scale for qualitative analysis

Grading	Image quality	Vascular contrast	Artefacts	Diagnostic confidence
*1*	Evident limitations in the vessel/wall/lumen definition due to strong image noise	Homogeneous contrast throughout all vessel sections	Present and affecting image evaluation	Unacceptable
*2*	Good, with minimal limitations in the vessel wall/lumen definition due to moderate image noise	Good contrast—Good distribution of the contrast agent and contrasting of the vascular course	Present and affecting vascular structures	Suboptimal
*3*	Very good, with well-preserved vessel wall definition and well-preserved delineated contour due to minimal image noise	Average contrast—Partially inhomogeneous distribution and contrasting of the vascular course	Present but not affecting visualization of vascular structures	Average
*4*	Excellent, with sharp vessel wall and well-delineated vessel contours due to poor image noise	Poor contrast—Distribution of the contrast agent and contrasting of the vascular course	None	Above average

**Table 2:** Cerebral vessel score

Vessel score	Description
*4—excellent*	Sharp vessel wall and well-delineated contour of the vessel
*3—very good*	Well-preserved vessel wall definition and well-preserved delineated contour
*2—good*	Minimal limitations in the vessel wall/lumen definition
*1—limited*	Evident limitations in the vessel/wall/lumen definition with diagnostic influence

### Quantitative Evaluation

For quantitative analysis, a board certified radiologist (blinded data, with 5 years of experience in neuroradiology) exported images from the Vitrea workstation (Canon Medical Systems, Otawara) as DICOM formats to a workstation including a MATLAB tool (R2017b, The MathWorks, Natick, MA).

The measurement of the steepness of gray value transition was performed in two vessel sections: extracranial ICA and intracranial basilar arteries (BA). Due to reproducibility and comparability reasons, measurements were taken exactly on the same anatomical vessel position. In the presence of hemodynamically relevant stenoses, calcified plaques, compromising surrounding processes or occlusion of the examined vessel, the contralateral side was used. Objective image quality parameters for examiner-independent assessment of contrast and delineation were determined as follows: three different parameters were gathered automatically, i.e., analysis of the distance between 25% and 75% of the maximum grey value in mm, difference in HU and steepest slope between the vessel and the perivascular tissue.

We selected an image region containing the vessel of interest, and thereby, the vessel region and other tissue types were automatically detected due to their high HU. The vessel region center was thus calculated, and a profile was drawn from that center through each voxel mapping to the vessel region border. Afterwards, the upper and lower baseline signal intensities (SI) as well as their 25% SI and 75% SI and their corresponding profile positions were determined for each profile. Furthermore, the slope of the transition between gray values inside and outside the vessel were calculated using linear regression. In order to reduce partial volume effects, only median parameters of the profiles showing 20% of the steepest slope values were processed.

To quantitatively evaluate additional image quality parameters, noise, SNR and CNR were analyzed in the ICA and BA.

The determination of the local noise in the immediate vicinity of the evaluated vessel was based on previous reports [9, 10] and hence we aimed at the determination of noise that remains unaffected by tissue structures or gray value trends. Consequently, a sliding window of 5 mm × 5 mm was applied to the previously selected ROI. If tissue transitions were in the actual position of the sliding window, the respective position was excluded, and the window moved to the next position. If no tissue transitions were included, the region was detrended by a 2D-polynomial fit of second order [11] and the standard deviation of the gray values was calculated. The minimum standard deviation of the complete ROI of all window positions was used for further SNR and CNR calculations.

**Fig. 2:**
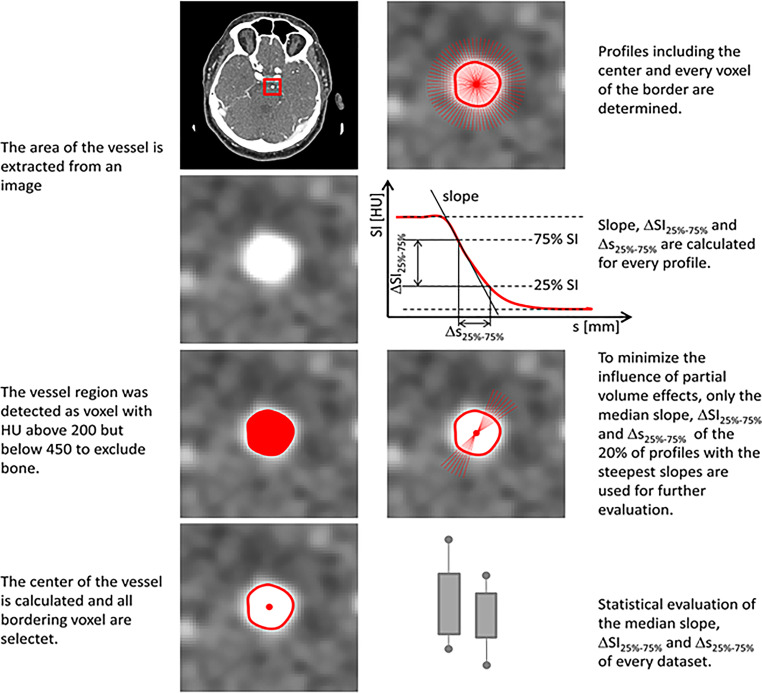
Process of quantitative analysis

The SNR was calculated as the signal intensity of the upper baseline, divided by the noise value, the CNR as the difference of the signal intensity of the upper and lower baseline, divided by the noise value. The quantitative analysis is illustrated in Fig. 2.

### Radiation Dose

CDTIvol and dose length product (DLP) were reported and effective dose was estimated by multiplying DLP with the conversion factor for neck-brain CTA (0.0031 mSv *mGy^-1^ *cm^-1^; adapted for CTDIvol values of a 16 cm head phantom) [10].

### Statistical Analysis

Statistical analysis as well as the illustrations were carried out using R 4.1.3 (R Foundation for Statistical Computing, Vienna, Austria).

Mean and standard deviation are reported for continuous variables. Median and frequencies are reported for ordinal variables. Student’s t‑test was conducted for comparing continuous variables and Wilcoxon-signed rank test for comparing ordinal variables. A two-tailed *p*-value of < 0.05 was considered statistically significant.

Interobserver variability was determined based on Fleiss-kappa. Kappa values of up to 0.20 were considered as slight agreement, 0.21–0.30 as poor agreement, 0.31–0.40 as fair agreement, 0.41–0.50 as moderate agreement, 0.51–0.70 as good agreement and > 0.7 as almost perfect agreement.

## Results

### Patient Characteristics

We identified 177 patients to be eligible for inclusion in the study but 20 patients were excluded due to severe artifacts (movement or beam hardening), insufficient contrast or due to venous contamination, leading to a final study cohort of *n* = 155 (M = 64; F = 91; age = 67.2 ± 11.3 years). The two sub-cohorts consisted of NR-CT: *n* = 73 (F = 49; M = 24) and UHR-CT *n* = 82 (F = 42; M = 40). Of the patients in the NR-CTA data set 12 were excluded (3 patients = severe metal artifacts; 1 patient = severe motion artifact and 8 patients = insufficient arterial contrast) and 8 patients of the UHR-CTA data set were excluded (7 patients due to insufficient arterial contrast and 1 patient due to metal artifacts).

### Qualitative Evaluation

**Fig. 3:**
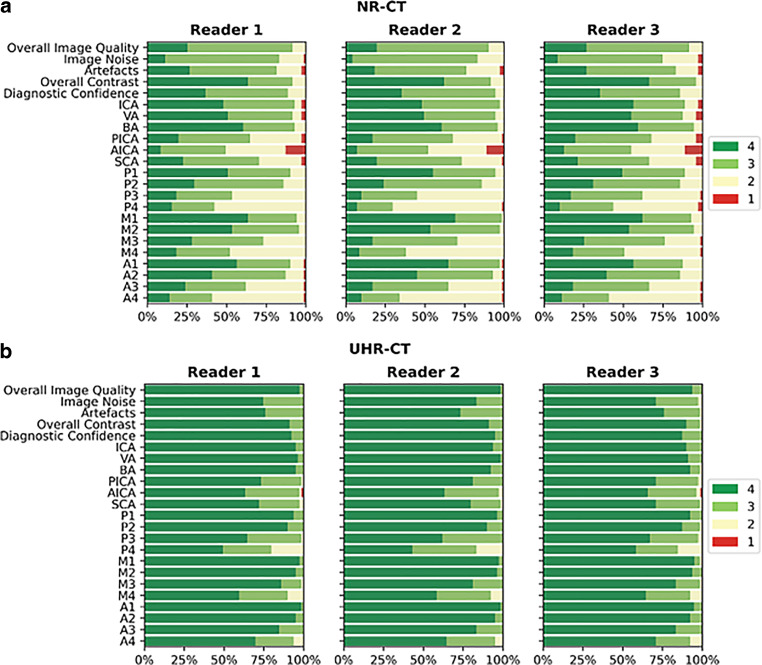
Bar charts of the Likert scale values and their distribution for CTA overall image quality, overall contrast, noise, artifacts, diagnostic confidence and the different vessels and vessel subsegments. UHR-CTA (**b**) and NR-CTA (**a**)

In comparison to NR-CTA, UHR-CTA showed significantly superior overall image quality. Briefly, UHR-CTA revealed higher image quality (*p* < 0.001), lower subjective image noise (*p* < 0.001), less artifacts (*p* < 0.001), higher overall contrast (*p* < 0.001) and higher diagnostic confidence (*p* < 0.001). Dedicated vessel evaluation showed superior neurovascular delineation and visualization of all vessel segments from proximal to distal for UHR-CTA compared to NR-CTA (PICA: UHR-CTA = 4 (3–4) vs NR-CTA = 3 (2–3); P4: UHR-CTA = 4 (3–4) vs NR-CTA = 2 (2–3); M4: UHR-CTA = 4 (4–4) vs NR-CTA = 3 (2–3); A4: UHR-CTA = 4 (3–4) vs NR-CTA = 2 (2–3); all *p* < 0.001). Medians and frequencies of the subjective assessment are demonstrated in Fig. 3 and Table 3. Interreader agreement between the three readers was good to almost perfect for image quality evaluation (kappa: 0.7, 0.6, 0.7 and 0.7 for artifacts, image noise, overall image quality and overall, respectively) and good for evaluating the delineation of the small, intracerebral arteries (kappa: 0.7).

**Table 3:** Quality statistics across raters: Compared medians, interquartile range and *p*-values

Metric	NR-CT median (IQR)	UHR-CT median (IQR)	*P* value
Overall image quality	3 (3–3)	4 (4–4)	*p* < 0.001
Noise	3 (3–3)	4 (3–4)	*p* < 0.001
Artefacts	3 (3–3)	4 (4–4)	*p* < 0.001
Overall contrast	4 (3–4)	4 (4–4)	*p* < 0.001
Diagnostic confidence	3 (3–4)	4 (4–4)	*p* < 0.001
Carotid arteries	4 (3–4)	4 (4–4)	*p* < 0.001
Vertebral arteries	3 (3–4)	4 (4–4)	*p* < 0.001
Basilar artery	4 (3–4)	4 (4–4)	*p* < 0.001
PICA	3 (2–3)	4 (4–4)	*p* < 0.001
AICA	3 (2–3)	4 (3–4)	*p* < 0.001
SCA	3 (2–3)	3 (3–4)	*p* < 0.001
P1	4 (3–4)	4 (4–4)	*p* < 0.001
P2	3 (3–4)	4 (4–4)	*p* < 0.001
P3	3 (2–3)	4 (3–4)	*p* < 0.001
P4	2 (2–3)	4 (3–4)	*p* < 0.001
M1	4 (3–4)	4 (4–4)	*p* < 0.001
M2	3 (3–4)	4 (4–4)	*p* < 0.001
M3	3 (3–4)	4 (3–4)	*p* < 0.001
M4	3 (2–3)	4 (3–4)	*p* < 0.001
A1	4 (3–4)	4 (4–4)	*p* < 0.001
A2	3 (3–4)	4 (4–4)	*p* < 0.001
A3	3 (2–3)	4 (4–4)	*p* < 0.001
A4	2 (2–3)	4 (3–4)	*p* < 0.001

*PICA* posterior inferior cerebellar artery, *AICA* anterior inferior cerebellar artery, *SCA* superior cerebellar artery, *P1-4* posterior cerebral artery sub-segments, *M1-4* middle cerebral artery sub-segments, *A1-4* anterior cerebral artery sub-segments, *IQR* interquartile range, *UHR-CT* ultra-high-resolution computed tomography, *NR-CT* normal resolution computed tomography

**Fig. 4:**
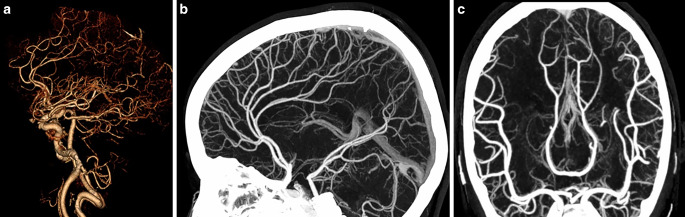
Example of a UHR-CTA of a female 41-year-old patient. **a** Volume rendering technique in the sagittal plane. **b** Maximum intensity projection with a slice thickness of 17.5 mm in sagittal view; **c** Maximum intensity projection with a slice thickness of 17.5 mm in Towne view for tracing the vascular course of the posterior cerebral artery. The superior image quality and the distinct delineation of the peripheral sub-segments are emphasized

**Fig. 5:**
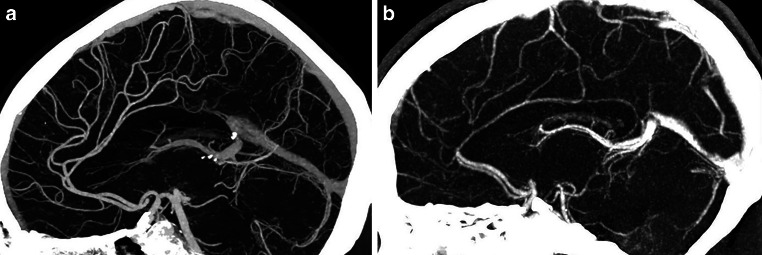
Two exemplary patients with the same clinical characteristics. **a** Maximum intensity projection with a slice thickness of 17.5 mm calculated on an UHR-CTA data set in sagittal view. **b** Maximum intensity projection with a slice thickness of 17.5 mm calculated on an NR-CTA data set in sagittal view. In contrast to NR-CTA, the significantly improved visualization of the small vessel branches in UHR-CTA can be considerably emphasized

A clear depiction and delineation of small, peripheral vessels in a patient example is given in Fig. 4, and the remarkable superiority of UHR-CT in the visualization of peripheral and small vessel branches and their delineation is demonstrated in volume rendering (Fig. 5). The improved image quality of the vertebrobasilar area is also substantial: VA: UHR-CTA = 4 (4–4) vs NR-CTA = 3 (3–4) PICA: UHR-CTA = 4 (3–4) vs NR-CTA = 3 (2–3); AICA: UHR-CTA = 4 (3–4) vs NR-CTA = 3 (2–3). The apparent differences in delineation of peripheral cerebral vessels are illustrated in Fig. 5.

### Quantitative Evaluation

**Fig. 6:**
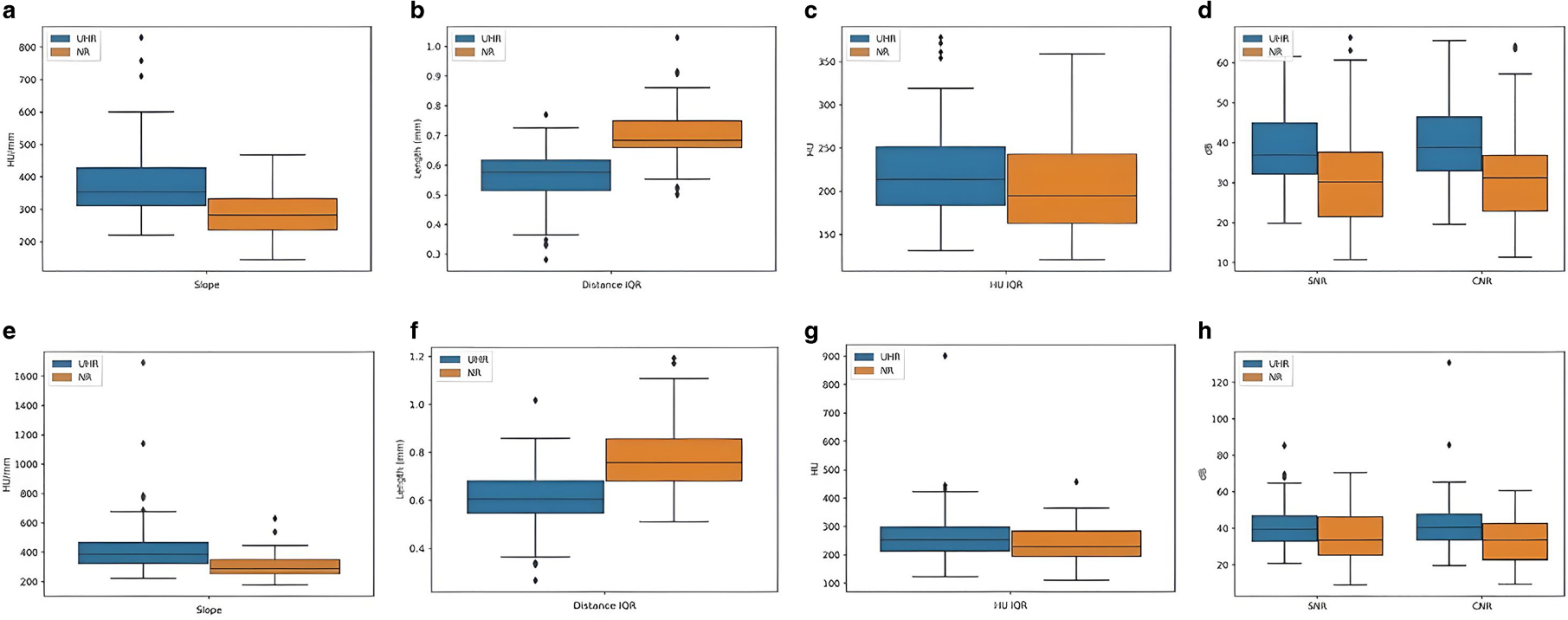
Value distribution of quantitative analysis. **a**–**d** Value distribution for the basilar artery. **a** Slope for UHR-CT and NR-CT in mm/HU. **b** Distance between 25–75% in mm. **c** Difference between 25–75% in HU. **d** SNR and CNR. **e**–**h** Value distribution for the internal carotid artery. **e** Slope for UHR-CT and NR-CT in mm/HU. **f** Distance between 25–75% in mm. **g** Difference between 25–75% in HU. **h** signal-to-noise ratio (SNR) and contrast-to-noise ratio (CNR)

The SNR and CNR were calculated for two types of arteries (ICA, BA) per patient, and their distributions were illustrated using a boxplot graph (Fig. 6). UHR-CTA showed significantly increased SNR (from NR-CT = 32.04 to UHR-CT = 38.20; *p* < 0.001) and CNR (from NR-CT = 31.3 to UHR-CT = 39.2; *p* < 0.001).

The slope of gray value transitions between intravascular and perivascular fibrous tissue for the internal carotid artery was steeper and increased (NR-CT = 307.2 HU/mm and UHR-CT = 432.4 HU/mm; *p* < 0.001). Consequently, the difference between 25% and 75% in HU was also increased (NR-CTA = 290.3 HU and UHR-CTA = 378.9 HU, *p* < 0.02), as well as the corresponding slope for the basilar artery (NR-CT = 307.2 HU/mm and UHR-CT = 378.4 HU/mm; *p* < 0.001) and the corresponding difference (NR-CT = 206.2 HU and UHR-CT = 251.3 HU; *p* < 0.02). All aforementioned metrics were analyzed and illustrated as boxplot graphs for BA and ICA (Fig. 6).

### Radiation Dose

The CTDIvol of NR-CT was 57 ± 7.2 mGy and the DLP was 563.2 ± 115.7 mGy*cm, resulting in a mean effective dose of 1.9 ± 0.3 mSv. In contrast, the mean CDTIvol of UHR-CT was significantly lower with 12.17 ± 4.36 mGy, the DLP was at an average lower with 486.3 ± 138.3 mGy*cm, which resulted in a mean effective dose of 1.5 ± 0.3 mSv. Radiation dose was significantly lower for UHR-CTA as compared to NR-CT (*p* < 0.001).

## Discussion

The purpose of our study was to assess the image quality and dose exposure of the UHR-CTA compared to NR-CT and to evaluate the potential benefit for routine application.

First technical reports have already indicated feasibility of UHR-CTA [12, 13], albeit without a comparative patient cohort study. The additional value of UHR-CT has been reported for studies focusing on other body regions, and its improved diagnostic ability to visualize even very small structures such as coronary arteries [14], the artery of Adamkiewicz [15, 16] or collaterals in moyamoya disease [17] has been highlighted.

The relevance of UHR imaging in clinical routine is expanding due to the implementation of two novel detector technologies, which provide ultra-high-resolution images (photon counting CT technology and UHR-CT with superfine detector elements). In particular, ultra-high-resolution vascular imaging bears new perspectives and is consistently in the focus of current research and frequently discussed [2, 3, 18].

The results of this study show that UHR-CTA provides excellent subjective and objective image quality as well as excellent visualization of small vascular and perivascular tissues and thus is superior to NR-CT. Data analysis yielded higher SNR and CNR as well as contour sharpness for UHR-CT. Although the spatial resolution and image quality were higher, UHR-CT was associated with a slight but significantly lower radiation dose compared to NR-CT. The reduction in radiation dose may be attributed to the new UHR detector system with a relatively low electronic noise [19], despite its superior resolution.

Due to a remarkably increased image quality in UHR-CTA, the identification of small vascular lesions is facilitated, and the resulting images simultaneously present with high diagnostic content and low noise. The qualitative and diagnostic improvement may be of value in many clinical situations. The high image quality and diagnostic confidence of UHR datasets could also contribute to a more accurate description of the configuration and to the detection of diminutive abnormalities, such as calcifications, daughter aneurysms, intranidal aneurysms and therapeutically relevant branching vessels. It may allow a clear distinction between intradurak and extradural localization of aneurysms; however, even with greatly improved image quality digital subtraction angiography still remains the gold standard in cerebrovascular imaging. Whereas increasing image resolution and improvements in SNR normally results in an increase of radiation doses, the UHR-CT system used in the underlying study provides lower radiation than the hitherto used NR-CT system, despite improved image quality. In line with the ALARA (as low as reasonably achievable) principle the use of UHR-CTA may enable an improved noninvasive, preinterventional planning. A precise planning based on this type of CT data may contribute to preferred diagnostic projections and may lead to shorter fluoroscopy times in diagnostic subtraction angiography. Additionally, patients would also benefit from a reduced radiation exposure. Ultra-high-resolution imaging of the cerebral vessels was achieved with 7T TOF MRA [20–22] and cone beam CT [23] with very promising results, allowing better visualization of the small and peripheral vessels and their perivascular tissue.

Furthermore, follow-up imaging after intracranial stenting may benefit from increased resolution and image quality. Another advantage of UHR-CTA may be a more precise evaluation of postinterventional, extracranial stent positions and the follow-up imaging. In particular, carotid stent and carotid endarterectomy monitoring may be facilitated, and complications such as intimal proliferation and restenosis of stents may be identified earlier, resulting in a quicker therapeutic response. This should be considered in studies combining UHR-CTAs with metal artefact reduction algorithms using homogeneous cohorts of stent-treated patients.

The advancement of clinical material science in mechanical thrombectomy may lead to an expansion of treatment indications in peripheral arterial occlusions in the future and thus, the here described improved delineation of vascular subsegments, especially the peripheral subsegments (M3, M4, P2, P3, and P4) may render these structures more accessible for both diagnosis and treatment [24, 25]. These results of improved visualization of peripheral vessel segments may appear particularly relevant in the context of current multicenter randomized treatment trials for distal thrombectomy.

Improved imaging, particularly of the vertebrobasilar arteries and cerebellar arteries, can facilitate the diagnosis of potentially life-threatening occlusions of these vessels and of diagnostically challenging vascular diseases such as Wallenberg syndrome.

The use of UHR-CT may enable the timely diagnosis of early stages of vascular aging, characterized by fatty streaks that accumulate during of the molecular pathomechanism in atherosclerosis. Hence, atherosclerotic plaques may be defined more accurately, resulting in a precise graduation of vessel stenosis. In particular, the differentiation of subtotal stenosis from vessel occlusion may be optimized, hereby aiding treatment decisions.

In addition, small vessel changes such as intimal flaps, arterial fenestrations, fibromuscular dysplasia or carotid webs could become amenable to diagnosis in CT imaging. Summing up, our results confirm the value of the UHR technique in clinical use and may also excite further pathology-associated studies and a comparative to evaluate the envisaged improvement.

We were limited by the retrospective study design and the small number of subjects including a possible selection bias. The lacking intraindividual comparison between NR-CTA and UHR-CTA is a further limitation of this study. The potentially obvious distinction between the image data from the new device and the old CT system could have caused reader bias, which we attempted to counteract with a randomized procedure and initial consensus training. Furthermore, our study lacks homogeneous pathological groups which represents a further limitation. Beyond that, the small sample size does not allow a generalization of our conclusions yet, and therefore, future prospective studies with larger cohorts are needed.

## Conclusion

Ultra-high-resolution CT angiography improves image quality in neurovascular imaging allowing the depiction and evaluation of small peripheral cerebral arteries. It may thus improve the detection of pathologies in small cerebrovascular lesions and the resulting diagnosis.

## Abbreviations

ICA: Internal carotid artery
AICA: Anterior inferior cerebellar artery
CNR: Contrast to noise ratio
CT: Computed tomography
CTA: Computed tomography angiography
CTDIvol: Computed tomography dose index
DLP: Dose length product
MCA: Middle cerebral artery
NR-CT: Normal resolution computed tomography
PICA: Posterior inferior cerebellar artery
ROI: Region of interest
SCA: Superior cerebellar artery
SNR: Signal to noise ratio
UHR: Ultra-high-resolution

## References

[1] Oostveen LJ (2020). Physical evaluation of an ultra-high-resolution CT scanner. Eur Radiol.

[2] Mergen V (2022). Ultra-high-resolution coronary CT angiography with photon-counting detector CT: feasibility and image characterization. Invest Radiol.

[3] Symons R (2018). Photon-counting CT for vascular imaging of the head and neck: first in vivo human results. Invest Radiol.

[4] Ohara A (2020). Improved image quality of temporal bone CT with an ultrahigh-resolution CT scanner: clinical pilot studies. Jpn J Radiol.

[5] Latina J (2021). Ultra-high-resolution coronary CT angiography for assessment of patients with severe coronary artery calcification: initial experience. Radiol Cardiothorac Imaging.

[6] Motoyama S (2018). Ultra-high-resolution computed tomography angiography for assessment of coronary artery stenosis. Circ J.

[7] Shanbhag SM (2019). Prototype ultrahigh-resolution computed tomography for chest imaging: initial human experience. J Comput Assist Tomogr.

[8] Ohno Y (2022). Comparison of lung CT number and airway dimension evaluation capabilities of ultra-high-resolution CT, using different scan modes and reconstruction methods including deep learning reconstruction, with those of multi-detector CT in a QIBA phantom study. Eur Radiol.

[9] Anam C (2021). An improved method of automated noise measurement system in CT images. J Biomed Phys Eng.

[10] Chun M, Choi YH, Kim JH (2015). Automated measurement of CT noise in patient images with a novel structure coherence feature. Phys Med Biol.

[11] Dobbins JT (2006). Intercomparison of methods for image quality characterization. II. Noise power spectruma. Med Phys.

[12] Ucar FA (2021). Feasibility of ultra-high resolution supra-aortic CT angiography: an assessment of diagnostic image quality and radiation dose. Tomography.

[13] Ogawa K (2021). Visualization of small visceral arteries on abdominal CT angiography using ultra-high-resolution CT scanner. Jpn J Radiol.

[14] Shanbhag SM, Chen MY (2021). Ultra-high-resolution coronary CT angiography: the “final frontier”—are we there yet?. Radiol Cardiothorac Imaging.

[15] Hino T (2020). Detectability of the artery of Adamkiewicz on computed tomography angiography of the aorta by using ultra-high-resolution computed tomography. Jpn J Radiol.

[16] Yoshioka K (2018). Ultra-high-resolution CT angiography of the artery of Adamkiewicz: a feasibility study. Neuroradiology.

[17] Fukushima Y (2022). Evaluation of moyamoya disease in CT angiography using ultra-high-resolution computed tomography: application of deep learning reconstruction. Eur J Radiol.

[18] Dangelmaier J (2018). Experimental feasibility of spectral photon-counting computed tomography with two contrast agents for the detection of endoleaks following endovascular aortic repair. Eur Radiol.

[19] Boedeker K (2018). Aquilion Precision ultra-high resolution CT:quantifying diagnostic image quality.

[20] Grochowski C, Staśkiewicz G (2017). Ultra high field TOF-MRA: a method to visualize small cerebral vessels. 7T TOF-MRA sequence parameters on different MRI scanners—literature review. Neurol Neurochir Pol.

[21] Harteveld AA (2016). 7-T MRI in cerebrovascular diseases: challenges to overcome and initial results. Top Magn Reson Imaging.

[22] Kraff O (2015). MRI at 7 Tesla and above: demonstrated and potential capabilities. J Magn Reson Imaging.

[23] Simoni A (2023). Innovative tool for automatic detection of arterial stenosis on cone beam computed tomography. Appl Sci.

[24] Stampfl S (2016). Initial experience with a new distal intermediate and aspiration catheter in the treatment of acute ischemic stroke: clinical safety and efficacy. J Neurointervent Surg.

[25] Kurre W (2017). Stent retriever thrombectomy of small caliber intracranial vessels using pREset LITE: safety and efficacy. Clin Neuroradiol.

